# Impaired decision-making in borderline personality disorder

**DOI:** 10.3389/fpsyg.2023.1109238

**Published:** 2023-08-04

**Authors:** Bettina Bajzát, Péter Soltész, Klára Soltész-Várhelyi, Evelyn Erika Lévay, Zsolt Szabolcs Unoka

**Affiliations:** ^1^Department of Psychiatry and Psychotherapy, Faculty of General Medicine, Semmelweis University, Budapest, Hungary; ^2^Department of General Psychology, Faculty of Humanities and Social Sciences, Pázmány Péter Catholic University, Budapest, Hungary

**Keywords:** decision-making, exclusion criteria based on compliance with the test, impulsivity, borderline personality disorders, neuropsychology, risk-taking

## Abstract

**Introduction:**

Borderline personality disorder (BPD) is a complex mental disorder with core symptoms like interpersonal instability, emotion dysregulation, self-harm, and impulsive decision-making. Previous neuropsychological studies have found impairment in the decision-making of patients with BPD related to impulsivity. In our study, we focus on a better, more nuanced understanding of impulsive decision-making in BPD with the help of Rogers’ decision-making test that simulates a gambling situation.

**Methods:**

A novelty of our study is that we excluded from further analysis non-compliant participants based on their performance. Outlier participants on the measures proportion of good choices and average of wager choice number were filtered out to represent the population that understood the basic premise of the task and showed minimal motivation to gain rewards. Thus participants often choosing the less likely color or frequently choosing the first bet amount available (to probably speed up the test) were omitted from further analysis. Another novelty is that we assessed and reported six variables that examine Deliberation Time, Quality of Decision, Risk-taking, Overall proportion bet, Delay aversion, and Risk adjustment. Forty-three women with BPD participated in the study, and 16 non-compliant were excluded. As for the healthy control group, 42 women participated in the study, and four non-compliant were excluded. Thus, we compared the data of 27 patients with BPD with 38 healthy controls.

**Results:**

Our results show that there are significant differences amongst the groups regarding the Quality of Decision Making (*F* (1,63) = 5.801, *p* = 0.019) and Risk Adjustment (*F* (1,63) = 6.522, *p* = 0.013). We also found significant interactions between group and winning probability regarding Risk Taking (*F* (4,252) = 4.765 *p* = 0.001) and Overall proportion of bets, i.e., the average proportion of bets relative to the total score of the subject (*F* (4,252) = 4.505, *p* = 0.002).

**Discussion:**

Our results show that the two groups use different decision-making strategies that can have various associations with everyday life situations.

## 1. Introduction

Disadvantageous choices are apparent in patients with borderline personality disorder (BPD). For example, they often engage in self-harming behavior and have difficulties in forms of substance abuse or binge eating problems. Impulsivity is one of the most dominant personality traits in BPD and plays an essential role in decision-making (Abraham and Nussbaum 2013).

Earlier research by Seres et al. (2009) investigated the relationship between impulsivity and neuropsychological dimensions in the case of BPD and found that neuropsychological deficits were significantly associated with impulsivity (Seres et al., 2009). Meta-analyses of BPD’s neuropsychological aspects show that BPD patients display various neuropsychological symptoms, namely deficits in attention, processing speed, cognitive flexibility, planning, visuospatial abilities, learning and memory skills, verbal intelligence, and decision-making (Ruocco, 2005; Unoka and Richman, 2016).

A recent meta-analysis by Paret et al. (2017) examined the results of studies comparing BPD patients’ decision-making with healthy participants. The analysis found significant differences in temporal delay discounting (TDD), in the Iowa Gambling Task (IGT) and other less frequently used decision-making paradigms.

As for TDD, BPD patients show a marked preference for smaller immediate rewards over delayed, more considerable gains (Paret et al., 2017). Previous research by Bechara et al. (1994) showed that decision-making deficits tested with the IGT are connected to impaired ventromedial prefrontal(VMC) /orbitofrontal(OFC) cortex functionality, and the amygdala also plays an important role in the decision-making process (Bechara et al., 1994, 1999, 2000; Damasio, 1998). Reduced amygdala volume (Nunes et al., 2009) and impaired OFC functioning have been identified in BPD patients (Carrasco et al., 2012; Krause-Utz et al., 2014), and in some cases, they show significantly worse performance on the IGT than healthy controls (Haaland and Landrø, 2007; LeGris et al., 2014), but meta-analysis also showed that sex, age, and medication significantly influenced IGT performance (Paret et al., 2017).

One of the less frequently used decision-making paradigms in BPD research is the one developed by Rogers et al. (1999). It is a neuropsychological task designed for further investigation of the decision-making deficits found in patients with OFC lesions and drug abusers. The main difference between the IGT and this task is that while the IGT requires the participant to find out the rules of the game (Bechara et al., 2000), in Rogers’ paradigm, the instructions and the probabilities of winning and losing are explicit, so the overall performance is less dependent on the learning component. Rogers’ paradigm examines the decision-making process from five main aspects: time of deliberation, quality of decision-making, willingness to take risks, ability to moderate risk as a function of the chance to win, and the task also make it possible to distinguish between impulsivity and risk-taking behavior (Rogers et al., 1999).

Brain areas that are involved in the decision-making process during the paradigm by Rogers, such as the dorsolateral prefrontal cortex or the anterior cingulate cortex (Yazdi et al., 2019) showed impairment in patients with BPD (Brunner et al., 2010; Sala et al., 2011; Krause-Utz et al., 2014; Lei et al., 2019). This would indicate the usefulness of this paradigm in this patient group. However, to date, not many studies have used this decision-making test in a BPD sample. Bazanis et al. (2002) found that patients with BPD performed significantly worse than the controls; it took them significantly longer to make their choices and presented marked effects of impulsivity on their decision-making (Bazanis et al., 2002). Kaplan et al. (2020) also used the paradigm to compare the decision-making of a BPD and a healthy control sample. However, they found no significant differences between the two groups (Kaplan et al., 2020).

Several other studies can justify the usage of this paradigm for the assessment of decision-making in BPD since they focused on difficulties that are also relevant to BPD. It was used in the study of alexithymia (Bibby, 2016), suicidal behavior (Gifuni et al., 2020), alcohol misuse (Harvanko et al., 2012; Gifuni et al., 2020), anxiety and mood disorders (Liaugaudaite et al., 2020), gambling disorder (Limbrick-Oldfield et al., 2020), decision making of opiate and amphetamine users (Psederska et al., 2021) and psychotic patients (Woodrow et al., 2019). Suicidal ideation and suicide attempters, Alcohol Use Disorder, and high alexithymia scores were associated with the fact that the participants placed higher bets even in uncertain situations. In contrast, this effect cannot be detected in heroin and amphetamine users compared to healthy individuals.

Besides exploring the differences in decision-making in BPD patients versus among healthy controls, we also aim to assess other possible variables that might have been overlooked in previous studies, such as the average proportion of bet relative to the current score, the tendency to impatiently accept larger percentages when the possible bets are presented in descending order, and the tendency to make larger bets on trials when the winning probability is larger and make smaller bets when the winning probability is smaller. Also, previous research did not cover the problem of non-compliant participants whose aim is not to perform as well as possible but rather to finish the task as quickly as possible, and for that reason consequently choose the first bet that is offered. This is an important issue since this behavior can be misinterpreted as impulsivity, which is the paradigm’s main focus. We addressed this by including only those participants in the final analysis whose performance indicated compliance with the paradigm’s instructions.

## 2. Methods

### 2.1. Subjects

The patient group consisted of 43 women with BPD who participated in a 4-week inpatient cognitive schema group therapy program at the Department of Psychiatry and Psychotherapy, Semmelweis University, Budapest, Hungary. All patients met the criteria for BPD according to the Hungarian version of the Structured Clinical Interview for DSM-IV Personality Disorders (SCID-II; Szádóczky et al., 2004). Exclusion criteria included the diagnosis of any neurological disorder with brain injury, intellectual disability, schizophrenia, and alcohol dependence or abuse two months prior to examination. Sixteen patients were excluded from the statistical analysis because of compliance issues.

The control group (CTRL) consisted of 42 healthy female participants and was matched with the patient group in terms of age and education. Control subjects had no history of psychotropic medication, and they completed the SCID-II and SCL-90 (Symptom Checklist-90-Revised; Derogatis, 1983) screening questionnaires. Four control participants were excluded from the statistical analyses because they did not follow the rules of the game.

All participants had Hungarian as their native language. Participation in the study was voluntary. Subjects gave written informed consent to participate in the study prior to the assessment, with no cash benefit. The local ethics committee of the Hungarian National Public Health and Medical Officer Service approved the study.

Characteristics of the patient and control groups are shown in Table 1, and there were no significant differences between the groups in terms of age and education.

**Table 1:** Demographic characteristics and medication of the compliant participants.

	Participants with BPD	Healthy control participants	Statistical tests
	(*n* = 27)	(*n* = 38)	
Age (years)	30.32 (±7.750)	26,87 (±6,775)	t(61) = 1.868; *p* = 0.067
Education			χ2 = 5,466; *p* = 0.486
Primary school	1	1	
Vocational school	1	1	
Secondary School	1	0	
Grammar school	12	14	
College	5	5	
University	5	16	
Postgraduate	0	1	
Number of BPD criteria* [SCID-II]	7.5	2.35	
Medication			
Antiepileptics	7	0	
Antidepressants	8	0	
Antipsychotics	10	0	
Anxiolytics	12	0	

* Number of yes on BPD module of the SCID-II screening questionnaire.

### 2.2. Decision-making task

**Figure 1:**
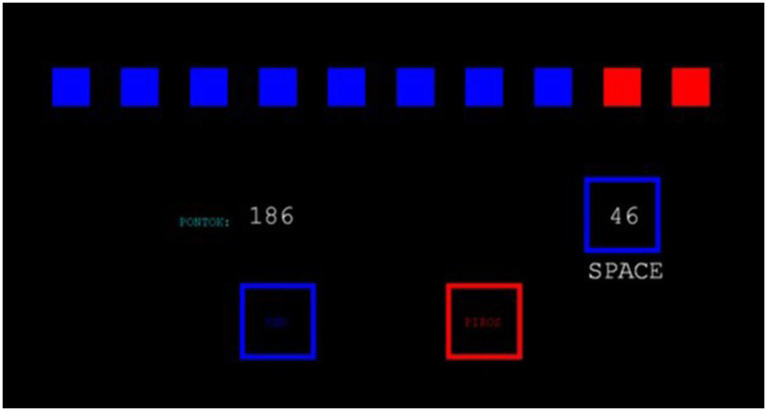
Screenshot of the Decision-making task. The blue-red ratio is visible at the top, the current score is shown in the middle left, and the current bet is in the blue square.

We used a computerized paradigm written in Matlab to assess the decision-making characteristics of the participants. A screenshot of the test can be seen in Figure 1.

The paradigm has eight blocks of trials. At the beginning of each block, the subjects get 100 points to start with. The goal is to collect as many points as they can in a block.

The paradigm’s each trial consists of two parts. In the first part, subjects have to make a simple probability-based decision. They can see ten boxes at the top of the screen; some are red, and some are blue. There is a yellow token under one of the boxes, and the subjects have to decide whether this token is under the red or the blue boxes. They can make this choice by using two buttons on the keyboard—the ratio of the red and blue boxes changes in each trial.

In the second part, after choosing the color, the subjects have to make a bet on that color. The computer generates the possible bets. In each trial, the computer usually offers five bets: 5, 25, 50, 75, and 95% of the subject’s latest score (except in those cases when the subject’s score is too low to get five offers, for example, when the subject has only 2 points). Each bet is displayed for 5 s; after the computer moves on to another bet, the subject cannot return to a previous one. The last offered bet gets automatically accepted if the subject does not respond. If the subject chooses the winning color, the bet is added to his/her latest score. If not, it is subtracted.

The first four blocks are in ascending condition. In these cases, the first bet is the lowest (5%), and the bets are increasing (25, 50, 75, and 95%). The other four blocks are in descending condition, the first bet is the largest (95%), and the bets are constantly decreasing (75, 50, 25, 5%).

### 2.3. Variables

The statistical analysis is centered around six main variables:

#### 2.3.1. Deliberation time (DT)

The time needed to decide which color to bet on.

#### 2.3.2. Quality of decision (QD)

The proportion of trials when the subject chose to bet on the more likely outcome.

#### 2.3.3. Risk-taking (RT)

The average proportions of the bets relative to the current score of the subject, considering only the cases when the subject chose the more likely outcome.

#### 2.3.4. Overall proportion bet (OPB)

The average proportion of bet relative to the current score.

#### 2.3.5. Delay aversion (DA)

The tendency to impatiently accept larger percentages when the possible bets are presented in descending order, but they can hold off betting in the ascending condition.

#### 2.3.6. Risk adjustment (RA)

The tendency to make larger bets on trials when the winning probability is larger and make smaller bets when the winning probability is smaller– considering only the cases when the subject chose the more likely outcome in the color decision phase.

### 2.4. Statistical analysis

We used one-way ANOVAs of the effect of group, and we used multiway ANOVAs, so that we can analyze the effect of group and the effect of winning probability and condition, and in some cases, we analyzed our data on a per choice base using Linear Mixed Models.

## 3. Results

### 3.1. Outlier detection

The decision-making task is a complex paradigm where subjects face risky choices similar to gambling situations. Completing the test was voluntary. All subjects had the opportunity to end the test at any time. The goal of the task was explicit: to collect as many points as possible. Subjects who adhered to the paradigm made choices that reflected their abilities and brought them closer to the goal. These compliant subjects showed variance in their choices of colors and bets based on the probabilities of winning. However, there were non-compliant subjects who did not operate within the boundaries of the task, i.e., some subjects either did not understand the importance of choosing the right color or bet to win more scores or did not find it interesting or important to win more scores. Either way, we think that it would be misleading to mix the data of these two types of subjects because the present article is mostly concerned about the choices made within the framework of the task.

We filtered our participants according to two criteria to exclude non-compliant subjects from the statistical analyses. We excluded those who had lower-than-expected logical color choices and those who, most of the time, chose the first bet presented to them regardless whether it was during ascending or descending condition of the test. Both of these cutoff points have been determined by k-means clustering to minimize the subjectivity of subject exclusion.

The first criterion is the proportion of good choices, where the cut point is determined by observing the histogram (~ 0.8 in this study). The second condition refers to the average of the wager choice number (the order number of chosen wager amount regardless of the ascending-descending conditions), the cut point is determined as previously (2.1).

**Figure 2:**
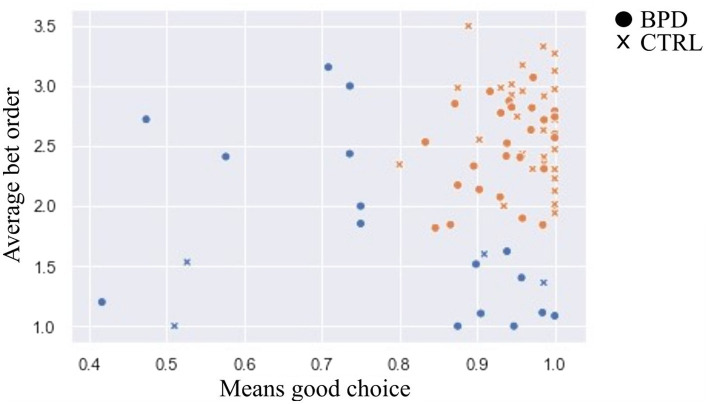
Outlier detection. Notes: X axis was the Means of good choice, where the cut point is 0.8, and the Y axis was the average bet order, where the cut point is 1.5. Sixteen persons who have been excluded are marked in blue.

As for the first criterion, we performed a k-means clustering algorithm on a single dimension: the percentage of logical color choices. Any color choice in a situation with five red boxes and five blue boxes counted as a logical choice. The cutoff point was determined by observing the histogram (Figure 2), and we defined it as around 0.8 (random choice would be around 0.6). This procedure led to the exclusion of 10 subjects (8 of them were patients with BPD).

As for the second criterion (i.e., unselective bet), we tested multiple k-means models on the previously filtered subjects with 2 to 5 clusters on the average bet order as a single dimension. (Note that the first bet was 5% of the current total score in the ascending condition, and it was 95% of the current total score in the descending condition).

Clustering into 3, 4, and 5 clusters resulted in very similar groups, with 1.5–1.6 as the average order number of the chosen bets. We chose clustering into 3 clusters because it led to the fewest excluded subjects. Thus, we excluded ten subjects (8 of them were patients with BPD).

Altogether, 20 subjects were excluded, and 16 were patients with BPD. Applying a Chi-square test showed that we excluded significantly more patients than healthy subjects. Altogether, 37.3% of BPD patients and 9.53% of CTRL subjects were excluded from the study.

### 3.2. Deliberation time (DT)

Using a two-way ANOVA, we analyzed the effect of the group and the effect of the winning probability on deliberation time (the time needed to decide which color to bet on). Although the patient group was slower than the control group, the main effect of the group was not significant (2,518 ms ± 1,131 vs. 2,289 ms ± 775; *F* (1,63) = 0.986, *p* = 0.325 Part. Eta^2^ = 0.015). Mean deliberation times were significantly longer at the less favorable ratios of red and blue boxes (e.g., 5:5) compared to the more favorable ratios (e.g., 9:1) [*F* (4,252) = 28.119, *p* = 0.000 Part. Eta^2^ = 0.309], but this increase did not differ significantly in the two groups [*F* (4,252) = 1.163, *p* = 0.328 Part. Eta^2^ = 0.018].

**Figure 3:**
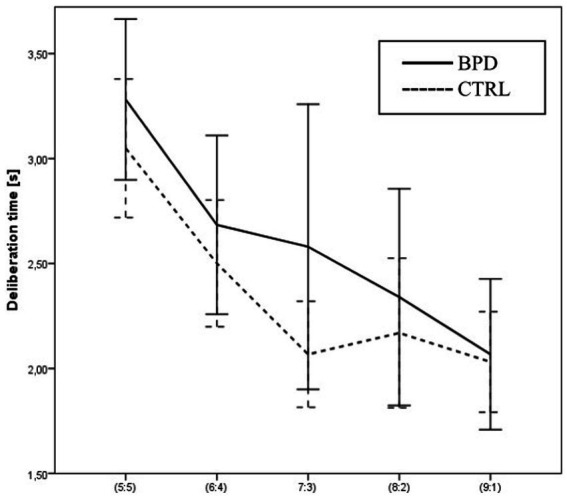
Differences in Deliberation times between BPD and CTRL groups along the probabilities of winning.

Using a one-way ANOVA, we analyzed the main effect of the condition on deliberation time. Mean deliberation times were not significantly different in ascending and descending conditions [*F* (1,63) = 1.486, *p* = 0.227 Part. Eta^2^ = 0.023]. The two-way interaction between the group and condition was also non-significant [*F* (1, 63) = 1.685, *p* = 0.199 Part. Eta^2^ = 0.026] (see Figure 3).

### 3.3. Quality of decision (QD)

The Quality of the decision reflects the proportion of trials when the subject chooses to bet on the more likely outcome, i.e., the color with the greater number of boxes. We analyzed the effect of the group and the effect of winning probability on QD with two-way ANOVA. The main effect of the group was significant. The BPD group’s performance was significantly worse than the control group’s performance [*F* (1,63) = 5.801, *p* = 0.019 Part. Eta^2^ = 0.084]. The QD increased significantly at more favorable ratios compared to less favorable ratios [*F* (3,189) = 16.485, *p* = 0.000 Part. Eta^2^ = 0.207]. However, the interaction between the group and winning probability was not significant [*F* (3,189) = 0.086, *p* = 0.968 Part. Eta^2^ = 0.001].

**Figure 4:**
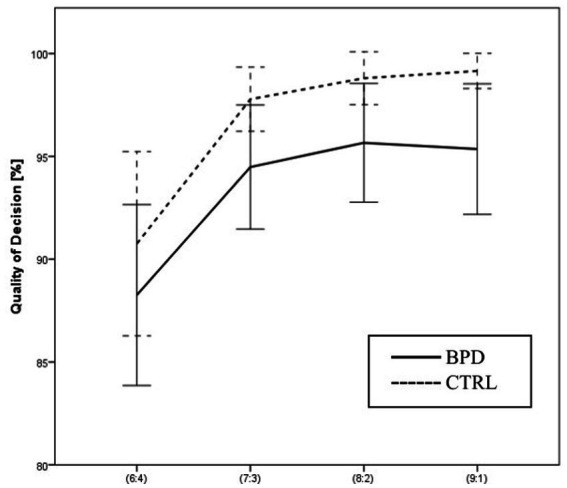
Differences in Quality of decision between BPD and CTRL groups along the probabilities of winning.

Both groups had a higher QD in the ascending condition than the descending condition, but the difference was not significant [*F* (1,63) = 0.972, *p* = 0.328 Part. Eta^2^ = 0.015]. The interaction between group and condition was not significant [*F* (1,63) = 0.164, *p* = 0.686 Part. Eta^2^ = 0.003] (see Figure 4).

### 3.4. Risk-taking

Using two-way ANOVA, we analyzed the effect of the group and the effect of the winning probability on RT, the average proportions of the bets relative to the current score of the subject. The main effect of the group was not significant [*F* (1, 63) = 0.154 *p* = 0.696 Part. Eta^2^ = 0.002]. The main effect of the winning probability on RT was significant [*F* (4,252) = 69.970 *p* = 0.000 Part. Eta^2^ = 0.526]. In both groups, the RT increased as a function of the ratio of red and blue boxes. The interaction between the group and winning probability was also significant [*F* (4,252) = 4.765 *p* = 0.001 Part. Eta^2^ = 0.070]. The BPD group is less risk-taking in the case of certain ratios of red and blue boxes, while they also take higher risks in the case of uncertain ratios than the CTRL group.

**Figure 5:**
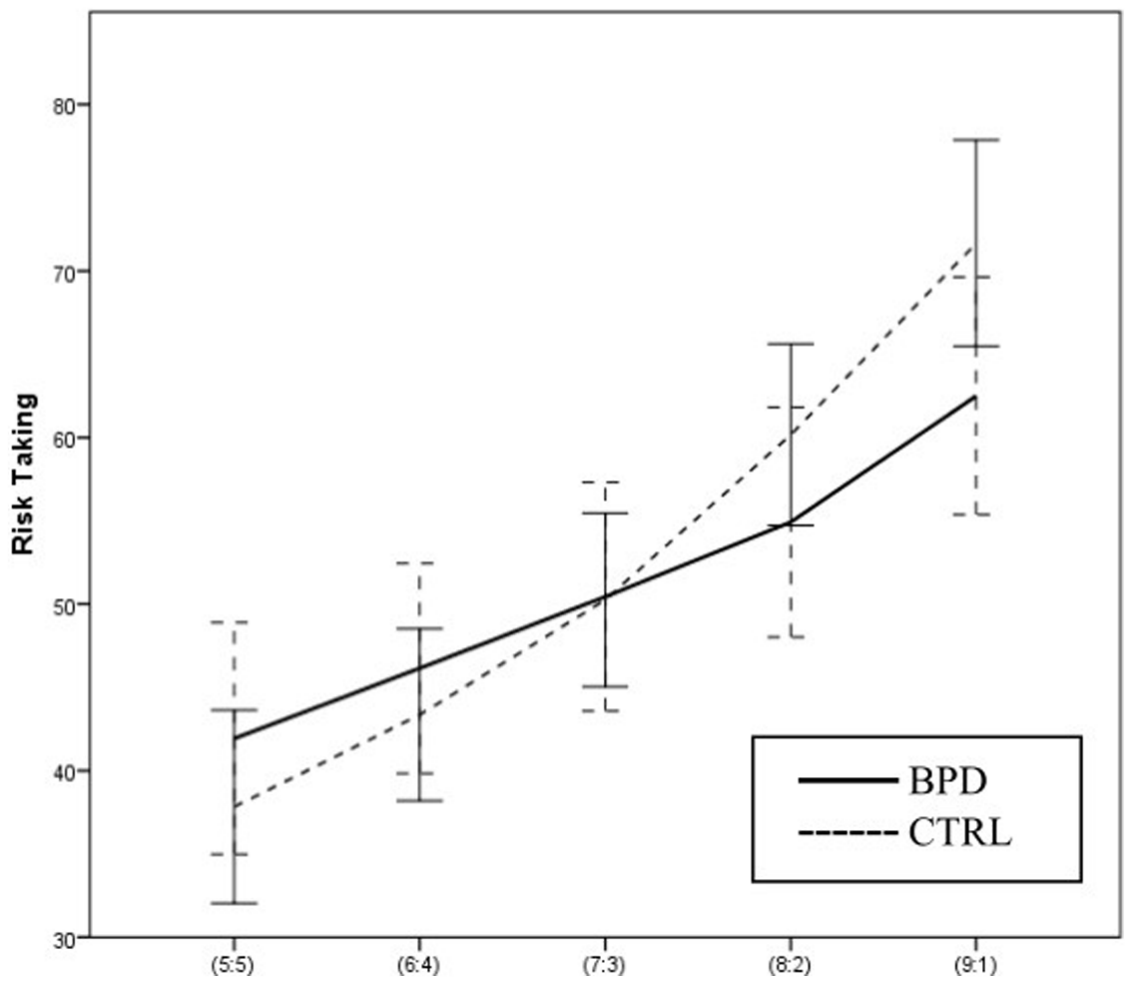
Differences in Risk taking between BPD and CTRL groups along the probabilities of winning.

Risk-taking was significantly different in the different conditions [*F* (1,63) = 69.282, *p* = 0.000 Part. Eta^2^ = 0.524], but the two-way interaction between group and condition was not significant [*F* (1, 63) = 2.718, *p* = 0.104 Part. Eta^2^ = 0.041] (see Figure 5).

### 3.5. Overall proportion Bet (OPB)

Using two-way ANOVA, we analyzed the effect of group and the effect of the winning probability on the overall proportion of bet, the average proportion of bets relative to the total score. The main effect of the group was not significant [*F* (1, 63) = 0.254 *p* = 0.616 Part. Eta^2^ = 0.004]. The main effect of the winning probability on OPB was significant [*F* (4,252) = 70.098 *p* = 0.000 Part. Eta^2^ = 0.527], and the interaction between the group and winning probability was also significant [*F* (4,252) = 4.505 *p* = 0.002 Part. Eta^2^ = 0.067].

**Figure 6:**
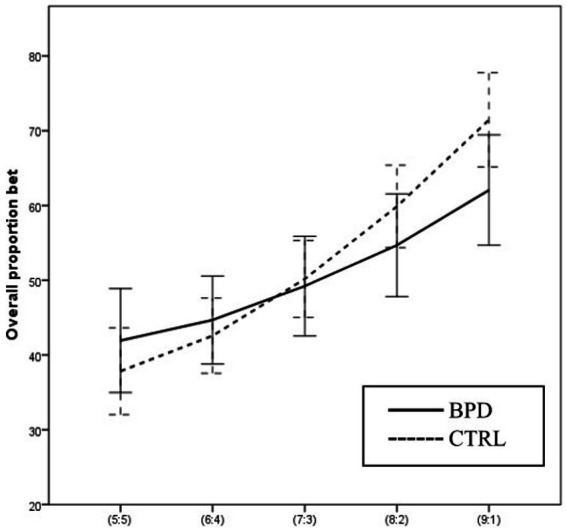
Differences in Overall proportion bet between BPD and CTRL groups along the probabilities of winning.

Overall proportion bet was significantly different in ascending conditions compared to descending conditions [*F* (1,63) = 79.593, *p* = 0.000 Part. Eta^2^ = 0.558], but the two-way interaction between group and condition was not significant [*F* (1, 63) = 1.981, *p* = 0.164 Part. Eta^2^ = 0.041]. The analysis of RT is almost identical to OPB (see Figure 6).

### 3.6. Delay aversion (DA)

Using two-way ANOVA, we analyzed the effect of group and the effect of the winning probability on DA, i.e., the tendency to bet immediate, larger percentages in the descending condition. The main effect of the group was not significant [*F* (1, 63) = 0.115 *p* = 0.735 Part. Eta^2^ = 0.002]. The main effect of the winning probability was significant [*F* (3, 189) = 37.889 *p* < 0.001 Part. Eta^2^ = 0.376]. DA was higher when the winning probability was higher. Using *Post hoc* analysis, we found that the 6:4 and 7:3 ratios did not differ from each other significantly (*p* = 0.068), but at the 8:2 ratio, DA was significantly higher than at both previous ratios (*p* < 0.001), and at the 9:1 ratio, DA was significantly higher than at 8:2 ratio (*p* < 0.001). The interaction between the group and winning probability was not significant (*F* (2, 189) = 2.539 *p* = 0.058 Part. Eta^2^ = 0.039).

Using Simple effects tests, we analyzed the differences between the four winning probability levels in the BPD and in the CTRL group separately. The effect of winning probability was significant both in the BPD [*F* (3, 61) = 22.103 *p* < 0.001 Part. Eta^2^ = 0.521], and in the CTRL group [*F* (3, 61) = 6.879 *p* < 0.001 Part. Eta^2^ = 0.253]. When the winning probability was higher, DA was higher in both groups. However, this finding was more emphasized in the BPD group. According to the *post hoc* analysis, in the BPD group, at all probability ratios, DA was significantly higher than at the smaller ratio. In the CTRL group, there was no significant difference between the 6:4 and 7:3 ratios, but at the 8:2 ratio, DA was significantly higher than at 7:3, and at 9:1, it was significantly higher than 8:2.

### 3.7. Risk adjustment (RA)

Using two-way ANOVA, we analyzed the effect of the group and the effect of the condition (whether the bets are presented in ascending or descending order) on RA, the tendency to adjust the bets to the winning probability. The main effect of the group was significant [*F* (1, 63) = 6.522; *p* = 0.013]. The RA was higher in the CTRL group compared to the BPD group. The main effect of the condition was also significant [*F* (1, 63) = 15.221; *p* < 0.001; Part. Eta^2^ = 0.195], and RA was higher in the ascending condition. The two-way interaction of group and condition was not significant [F(1, 63) = 2.697; *p* = 0.106; Part. Eta^2^ = 0.041].

The interaction of group and winning probability is not significant, [*F* (1, 63) = 0.131; *p* = 0.719; Part. Eta^2^ = 0.002].

**Figure 7:**
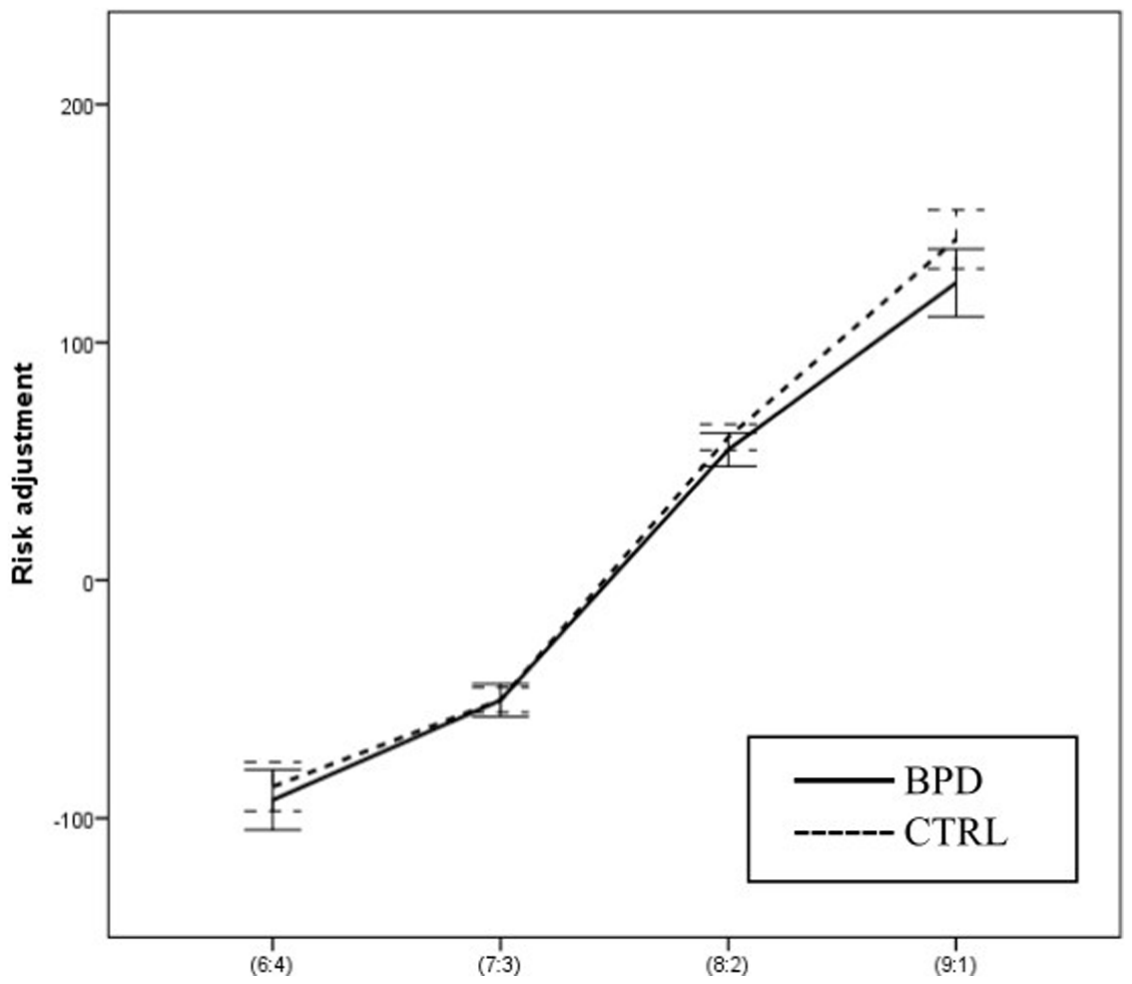
Differences in Risk adjustment between BPD and CTRL groups along the probabilities of winning.

Using 3-way ANOVA, we analyzed the effect of the group, the effect of the winning probability, and the effect of the condition. The main effect of the condition was significant; RA was higher when the bets were presented ascending/. The main effect of the winning probability was also significant. RA was higher when the probability of winning was higher. The main effect of the group was not significant. However, we found a significant interaction between the group and winning probability. According to the graphs, the interaction can be explained by the fact that the value of RA increases more steeply with an increasing probability of winning in the BPD group than among the controls. However, the effect is weak, so Simple effects testing can only partially confirm it. Using Simple effects tests, we analyzed the effect of the winning probability in the BPD group and the CTRL group separately. We found significant differences in both groups, but the size of the effect was larger in BPD [CTRL: *F* (3, 61) = 8.888 *p* < 0.001 Part Eta^2^ = 0.304; BPD: *F* (3, 61) = 34.700 *p* < 0.001 Part. Eta^2^ = 0.631] (see Figure 7).

## 4. Discussion

In our study, we examined the decision-making strategy of a group of patients with BPD and compared them to a group of healthy participants. We used a computerized decision-making task that we created based on the one developed by Rogers et al. (1999). Our goal was to assess and report all available variables, even the ones that might have been missed in previous literature. We also aimed to distinguish between participants who understood and followed the instructions of the paradigm and those who could not and applied this distinction as an additional exclusion criterion before the statistical analyses.

A significant proportion of BPD patients were non-compliant during the test, which may also indicate their impulsivity. This is because they chose the first response presented in every decision, regardless of the expected outcome. Two previous studies comparing people with BPD with healthy individuals have produced contradictory results, which may be explained by the fact that these studies did not exclude non-compliant individuals (Rogers et al., 1999; Bazanis et al., 2002).

We found no significant difference between the two groups with respect to Deliberation time. Previous studies found significant deviations regarding Deliberation time when examining patients with prefrontal cortex (PFC) lesions, such as” patients with subarachnoid hemorrhage of the anterior communicating artery” (Mavaddat et al., 2000), frontotemporal dementia (Rahman et al., 1999), and extensive prefrontal lesions that include the orbitofrontal cortex (Clark and Manes, 2004). Contrary to our findings, Bazanis et al. (2002) found a significant main effect of the group on deliberation time (Delay in the BPD group). They draw a conclusion similar to Rogers et al. (1999), who examined “patients with focal damage to the orbital as opposed to the dorsolateral or dorsomedial parts of the frontal lobes” and found that the two frontal patient groups differed in deliberation time, while the orbitofrontal patients slowed down like the drug users, the performance of the other frontal group was similar to the performance of the healthy group. Although deliberation times, in general, were significantly increased at the less favorable ratios, there was no significant interaction in the groups. Similar to our results regarding deliberation time, Bazanis et al. (2002) did not find a significant interaction between the groups and conditions (ascending or descending), either. The lack of significance in our results regarding the main effect group could be due to the exclusion of non-compliant participants, who appeared predominantly in the BPD group. All in all, our results do not show different deliberation times in the BPD group and in the healthy control group, which suggests different decision-making speeds among patients with BPD and patients with different frontal lobe lesions.

As for the Quality of the decision (the color choice), our results showed a significant main effect of the group. The BPD group performed significantly worse than the control group. This is in line with previous studies that examined men with gambling disorders and matched healthy participants (Limbrick-Oldfield et al., 2020) and individuals with alcohol problems (Harvanko et al., 2012) and found that the patients performed worse than the healthy controls. However, there are also contradictory results in the literature. For example, Psederska et al. (2021) examined opiate users and found no significant difference compared with healthy controls (Psederska et al., 2021).

We did not find significant differences between the groups with regard to the group’s main effect on Risk-taking and Overall proportion bet variables. However, when analyzing group and condition interaction, significant differences emerged: the two groups applied significantly different strategies at certain winning probabilities in the two conditions. Healthy participants choose larger bets in response to increasing winning probabilities, i.e., at a 5:5 ratio, they choose the smallest bet possible, and at a 9:1 ratio, they choose the largest bet possible. In contrast, participants with BPD risk more considerable amounts at smaller winning probabilities, while at larger winning probabilities, they are more reserved and choose smaller bets than healthy controls. This decision-making strategy of the BPD group is similar to that observed by (Liaugaudaite et al., 2020) in the study of individuals with suicidal thoughts, who also risk higher amounts in uncertain situations, but cannot do so in certain situations (Liaugaudaite et al., 2020).

As for delay aversion, we did not find any significant results regarding the main effect of groups or winning probability on delay aversion. Their interaction was not significant, either. With respect to winning probability, both groups show similar tendencies: they choose small bets when the winning probability is small and large bets when it is large. However, the difference between the bets made at large and small probabilities is smaller in the BPD group.

In the case of risk adjustment, we found that the condition –ascending or descending– has a significant main effect on the variable. In a previous study, they found that with the progression of age, the Quality of decision-making and Risk adjustment scores show a significant decline, which is similar in the case of patients with orbitofrontal cortex lesions (Woodrow et al., 2019), and in long-term amphetamine abuse (Rogers et al., 1999). By contrast, in the case of damaged dorsolateral prefrontal cortex, the Quality of decision is impaired while Risk adjustment is intact (Rogers et al., 1999). In depression Quality of decision is intact while Risk adjustment is impaired. Thus, it is presumable that the assessment of these variables in relation to each other could be an essential factor in the study of decision-making. As for individuals with BPD, we can say that they perform significantly worse than healthy controls regarding Risk adjustment and Quality of decision-making.

## 5. Limitations

A few important limitations need to be considered. First of all, we did not use self-report questionnaires in, which could explain the reason for such a high rate of non-compliance in the case of the BPD group. However, an important difference in the impulsivity of BPD patients appears to be that significantly more patients than healthy individuals failed to meet the task requirements.

Secondly, there was no detailed psychopathological examination of the participants, which would have determined the current level of depression and anxiety, or a third group of patients with different psychopathology, e.g., depression, so we can not say for sure that the found differences between groups are BPD-specific. Acute depressive and anxiety states can significantly affect performance and even cause non-compliance in participants.

Also, because of the exclusions, there is a possibility that the loss of statistical power led to non-significant results in the case of some variables, and that limits the interpretation of our results.

Furthermore, during the test recording, the ascending conditions came first in each case. It is possible that the differences between the two conditions were caused by fatigue during the test. In the future, we plan to present the ascending and descending conditions alternately so that we can see if the difference between the two conditions remains significant.

Finally, another limitation of the research is that the effects of psychiatric medications taken by the patients were not taken into account in the decision-making task. This was partly because we did not have enough data on the exact amounts of medication taken during the period of the test recruitment, and partly because it was not possible to create homogeneous groups for psychiatric medication taken by patients. A larger amount of patient data would have be needed to study the effect of the medication.

## 6. Conclusion

In our study, we used a decision-making task whose completion requires brain areas that show impairment in BPD to compare the decision-making strategy of a BPD group and a healthy control group. We excluded subjects who did not follow the rule of the decision-making game to make sure that we, in fact, compare the decision-making process of the groups purely without the noise of non-compliant behavior. Among the excluded individuals, there were significantly more patients with BPD, which may also be a characteristic feature of the disorder, i.e., non-compliance in certain situations. However, the assessment of this characteristic was not the object of our study. Thus, statistical analyses were only carried out with the compliant participants. Our results showed that patients with BPD made significantly more disadvantageous choices, while there was no difference in response time between the two groups. In other words, patients with BPD are more risk-takers when the probability of winning is low and taking less risk when the probability of winning is high, compared to healthy individuals. In conclusion, patients with BPD are prone to choose less efficient decision-making strategies, leading to poorer performance if they engage in the decision-making process at all. Ultimately, our results may contribute to a better understanding of how BPD patients differ from healthy individuals in terms of decision-making. It is clear that for patients it is more difficult adhering to the initial task instruction, and they handle certain and uncertain situations differently than healthy individuals. Patients with BPD are more insecure in uncertain situations and take more risks compared to healthy individuals. Similarly to their performance in the decision-making task, in therapeutic situations premature discontinuation of therapy or inadequate cooperation in therapy is a common problem for patients with BPD.

## Data availability statement

The datasets presented in this study can be found in online repositories. The names of the repository/repositories and accession number(s) can be found at: https://osf.io/d6kj3/.

## Ethics statement

The studies involving human participants were reviewed and approved by the Semmelweis University’s Regional, Institutional Scientific and Research Ethics Committee. The patients/participants provided their written informed consent to participate in this study.

## Author contributions

ZU and PS, designed the study and supervised the data collection. PS and BB analysed the data. BB, PS, KS, EL, and ZU participated in drafting and writing the manuscript, contributed to its revision, and take responsibility for the integrity of the data. All authors contributed to the article and approved the submitted version.

## Funding

ZU was also supported by the Hungarian National Research, Development and Innovation Fund (Grant no NKFI-132546).

## Conflict of interest

The authors declare that the research was conducted in the absence of any commercial or financial relationships that could be construed as a potential conflict of interest.

## Publisher’s note

All claims expressed in this article are solely those of the authors and do not necessarily represent those of their affiliated organizations, or those of the publisher, the editors and the reviewers. Any product that may be evaluated in this article, or claim that may be made by its manufacturer, is not guaranteed or endorsed by the publisher.
